# Liminal bioethics for liminal statuses: A new method for analysing novel biological entities

**DOI:** 10.1111/bioe.70064

**Published:** 2025-12-07

**Authors:** Michael Wee, Ilina Singh

**Affiliations:** Ethox Centre, University of Oxford, Big Data Institute, Old Road Campus, Oxford OX3 7LF, United Kingdom; Neuroscience, Ethics & Society (NEUROSEC), Department of Psychiatry, University of Oxford, Warneford Hospital Warneford Lane, Oxford OX3 7JX, United Kingdom

## Abstract

Novel biological entities such as cell lines and organoids do not typically fit into established conceptual categories, such as ‘human’ or ‘non-human’, ‘gift’ or ‘property’. This makes developing robust ethical principles or policy solutions difficult. In this article, we present a new approach to the ethics of novel biological entities, which we call ‘liminal bioethics’. We argue that some entities are best understood *as* liminal; we should not try to shoehorn them into existing categories or modify our concepts to suit them. However, we must investigate which concepts help articulate an entity’s liminal status most precisely. The choice of concepts, in turns, may suggest correspondingly liminal ethical principles or solutions for that entity. To demonstrate how this method works, we focus on immortalised cell lines as a test case, while also considering its implications for more recent breakthroughs in cell culture technology relating to organoids and assembloids. First, we demonstrate why the gift-property distinction is inadequate for framing the liminality of cell lines. We then argue for cell lines as being located in a liminal conceptual space between body part and organism, and discuss the conceptual shift that this entails for understanding the status of cell lines and organoids. We suggest that ethical principles in relation to cell lines should reflect this particular liminality; for example, commercial exchange of cell lines seem more acceptable than with body parts, but some means of respecting cell lines’ continuing human connection should still be in place.

## 1 Introduction

A recurring theme in the literature on novel biological entities, from cell lines to neural organoids, is that they disrupt or do not fit into established conceptual categories. Their liminality is of particular concern when they are created from human cells, since they raise privacy issues through the human DNA they contain, and they may be seen as a reflection or continuation of our biological lives and our human identity—as HeLa cells are sometimes portrayed in relation to Henrietta Lacks. Without firm categories of moral, ontological or even legal status to place them in, it can be difficult to develop robust and widely-accepted ethical principles for the use or creation of some novel biological entities.

In this article, we present a new approach to the ethical analysis of such novel biological entities, which we call ‘liminal bioethics’. In essence, we argue that it is sometimes necessary to accept some entities *as* liminal, especially where liminality is a persistent feature of an entity and is the direct cause of ethical difficulties surrounding it, rather than try and fit such entities into our categories or modify our concepts to suit them. Such entities are best understood as occupying a conceptual space between two well-established categories. This, in turn, should invite the development of ethical principles or solutions that are correspondingly liminal. We begin by outlining our approach, showing how it can contribute to ongoing bioethical discussion of novel entities. Then, we discuss the liminal status of immortalised cell lines, as a test case to demonstrate the conceptual analysis required for our approach. In this regard, we first argue that the gift-property framework, despite its dominance in the literature, is not suited for articulating the liminality of cell lines because the paradigm of property rights does not capture important socio-ethical aspects of the human body. We then argue for cell lines as being located in a liminal conceptual space between body part and organism, which we believe most precisely articulates the continuity and discontinuity between cell lines and human life and identity. We then discuss what kind of ethical principles and solutions can be developed on the basis of this particular liminality, before reflecting more generally on our method.

## 2 Liminality In Bioethics

Although the term ‘liminality’ is not frequently used in bioethics, it seems to capture something generally agreed upon about novel biological entities—they do not fit into our most well-established conceptual categories. Nonetheless there is some precedent for this use of ‘liminality’: the term has been used in relation to questioning how certain forms of medical innovation [1] or novel procedures such as bioprinting [2] disrupt the conventional distinction between medical research and treatment. Research and treatment are, of course, social practices, and these discussions of liminality tend to reflect the social dimension of practices that challenge the binary framing of research and treatment in healthcare regulation. Moss, for instance, focuses on the how bioprinting disrupts the conventional model of an intervention moving from research to treatment after sufficient population-level evidence, since the effectiveness of bioprinting is patient-specific and cannot be tested in a generalisable way as other interventions tend to be [1]. Sethi emphasises how a liminal approach to understanding medical innovation can help take into account the “different experiences of actors and processes” not captured by rigid categories of research and treatment [2, p. 125].

While recognising the importance of these preceding discussions of liminality in bioethics, our approach focuses on a different kind of liminality—one that is related to entities themselves, rather than regulatory classifications of social practices. Hence, in this section, we explain how liminality can be developed into a robust method for engaging with novel biological entities, which invoke different conceptual concerns from discussions of liminal practices between research and treatment.

### 2.1 Novel biological entities: Some conceptual challenges

Novel biological entities are a heterogenous class, comprising entities more routinely used in research today such as immortalised cell lines and induced pluripotent stem (IPS) cells, to newer or more controversial entities such as human-animal hybrids, embryoids, and organoids. Debates about nomenclature have been a consistent feature regarding such entities. Even in relation to cloning, where the resultant entity is not strictly novel, there have been attempts to resist using the term ‘embryo’, with suggestions like “clonote”, “SCNT-construct” and “embryoid blastocyst” being offered in place [3, p. 7]. More recently, Paşca et al. have provided a new framework for the nomenclature of multicellular models such as organoids and assembloids [4]. Notably, the authors unanimously reject as misleading terms like “mini-brain” and “brain-in-a-dish” which are often used in popular writing to describe neural organoids [4, p. 909]. It is an indication that language and the conceptual background we invoke matter when trying to understand such novel entities.

The importance of using the right linguistic concepts is reflected in the wider literature, where novel entities have been described as “inescapably hybrid, at once ‘life’ and ‘thing’” [5], as well as “neither human nor non-human” [6]. The liminality of these entities is thus a consistent theme. A number of new technical terms have been proposed to capture this liminality, each with different emphases, such as “bio-objects” [7] (emphasising the objectification of biological life), “sobjects” [8] (being both “subjects” and “objects”)^1^, and “person-related biological entities” [9] (the close relation to questions of personhood).

No doubt, this liminality makes ethical reflection on the use and creation of such entities particularly difficult, especially when liminality relates to human life and identity, broadly construed. But what these examples also make clear is that how we understand the liminality of new entities depends in large part on what categories we use to frame that liminality. The framing of novel biological entities as “between thing and person” [9], for example, directs attention towards ontological and moral status, with the concept of personhood as a reference point. By contrast, cell lines have generated a lot of commentary relating to consent and property rights. Although the validity of the gift-commodity distinction for biobanking ethics is increasingly being questioned [10], it has been a dominant framework in considering the status of cell lines and excised human tissue.

Evidently, our choice of concepts also makes a huge difference to how we address the ethical challenges posed by such entities. After all, that choice is typically not a matter of mere terminology, but already a normative question about which presuppositions or conceptual paradigms have explanatory power. Personhood comes with a conceptual appendage of consciousness and moral status, property with implications about ownership and pecuniary benefit.

Furthermore, when an entity of ambiguous status is framed by a particular set of concepts, it tends to invite one of two responses: Either we shoehorn the entity into one category or the other, or we modify the existing categories. Both responses are clearly seen in debates around property rights in the human body. Consent-based models for research on tissue samples and cell lines—the dominant model in American and British regulation—resist all or most claims to property rights in the biological material. They are to be treated primarily as gifts to the scientific enterprise. By contrast, others have argued that some modified understanding of property may be able to accommodate the status of human biological material. A more radical version of this second response would be to question whether the relevant conceptual categories no longer serve us well because they are socially constructed—as Holmberg et al. do in relation to species boundaries, when considering human-animal hybrids [11].

### 2.2 Liminality as an analytic method

*“Liminal entities are neither here nor there; they are betwixt and between the positions assigned and arrayed by law, custom, convention, and ceremonial.” – Victor Turner* [12, p. 95]

In response to these conceptual challenges, we believe that there is a third way: Some entities are best understood *as* liminal, and we should take this possibility seriously. But framing the entity’s liminality is no straightforward task, and necessarily involves robust analysis of which conceptual categories are more and less adequate. A liminal status also invites the possibility of developing correspondingly liminal ethical principles or solutions—which may be principles between two fixed ethical paradigms, or solutions which focus more on a continual process of dialogue and consultation among stakeholders rather than providing rigid policy solutions. We call this analytic approach ‘liminal bioethics’.

Our conception of liminality draws on earlier theoretical explorations of liminality, but is to be understood primarily as a method for bioethical analysis. Hence, we do not propose an overly taxing notion of liminality. Like Turner, the first major theorist of liminality—who followed van Gennep in investigating the liminal phase of ceremonies and rituals—we take the chief characteristic of liminality to be ambiguity in relation to two categories [12, 13]. In the context of Turner’s and van Gennep’s anthropological work, this ambiguity has an added connotation of being associated with the transition from one social or ritual phase to another. For our context, we take a broad view of what it means for an entity to be ambiguously located in between two well-established conceptual categories. It is possible that some entities appear to occupy both categories simultaneously, while others seem to occupy a persistently transitional phase between the two categories. What matters is the uncertainty associated with the in-betweenness of the liminal entity. It is important to distinguish liminality with mere hybridity—in the simple sense of having mixed elements—or from being a definable moderate or middle position—such as how political centrism is in the middle of left and right, but is not necessarily liminal even though its position is conceptually relative to the two ends of the spectrum. Genuine liminality is marked by the difficulty of even placing an entity firmly in between two categories. For example, we may not wish to call an entity of human biological origin ‘non-human’ or a mere ‘thing’, while recognising it is not a human being either. It is not a mere mix or middle position of the two categories, but something seemingly disruptive to the categories themselves.

Another feature of our conception of liminality is its persistence. The novel entities that are candidates for this liminality, we suggest, seem to resist neat categorisation even as we gain a more robust understanding of them and of the categories surrounding them. Their liminality is therefore not a mere accident, in consequence of our incomplete conceptual categories, but reflects some intrinsic ontological quality. Furthermore, each entity has to be understood as liminal on its own terms, and so a more precise conceptual framing is needed for each entity.

The most difficult aspect of liminal bioethics is identifying the most appropriate and precise conceptual framing of an entity’s liminality. As already suggested, we take the view that liminality is relative to the conceptual categories we are relying on, and this is grounded in a view of concepts as being informed by their paradigm cases and their theoretical presuppositions [14]. Some indeterminacy at the margins of even our most well-established and well-tested conceptual categories is inescapable, but this is not necessarily a bad thing—it is only when we have a pair of concepts in place that we can understand more precisely how a liminal entity is located ‘in between’ those concepts. Choosing which concepts to use or not is to assess both their explanatory power and which values are most stake with the entity in question. Hence, finding which concepts best frame an entity’s liminality is already a normative matter, and provides greater conceptual clarity to any ethical solutions that are proposed to cohere with that liminality.

In testing different framings of liminality, there are two pitfalls to be avoided. First, it is true that some amount of debate about the nature of categories is unavoidable in philosophy, jurisprudence and ethics. But it is sometimes the case that the appearance of liminality is generated by deeply entrenched debates about the categories themselves, in which case liminal bioethics may not be the best approach. For example, some might be tempted to treat brain death as a liminal state between life and death. But debates about brain death often turn on deeper disagreements about what is death in the first place.

Second, in order to do bioethics, the liminality in question should have clear normative implications. Viruses could be thought of as being on the threshold of chemistry and biology—in and of themselves, they do not exhibit organismic autonomy in the way we typically expect of biological entities, but when they do find a host, they behave in ways closer to the paradigm of biology than chemistry, particularly through replication. This account of liminality seems to be a conceptually sound and scientifically useful one, but it has little obvious significance for bioethics. It does not mean we must restrict ourselves to overtly normative categories like ‘moral status’, but it must be possible to draw normative implications from the categories we are using for how we ought to treat the entity in question.

With these considerations in place, we now turn to demonstrating the practical importance of liminality.

## 3 Locating Liminality

One area of biology where liminality is of increasing importance is field of cell culture and in particular the use of cellular and multicellular models. The discovery of HeLa cells in 1951 marked a turning point in cell culture, paving the way for more immortalised cell lines to be generated, thereby enabling many breakthroughs in research, especially in relation to drug and vaccine development. These cell lines were, nonetheless, 2D cell cultures and remained relatively limited in their ability to mimic in vivo biological processes. More recently, the development of organoids has marked the shift from 2D to 3D models, with organoids more closely resembling the tissue and functions of an organ, albeit on a smaller scale. Neural organoids have been of particular interest to researchers, as they offer the possibility of studying brain function without the need for in vivo testing. Neural organoids can be developed to mimic different stages of brain development, or could be modified with gene-editing tools like CRISPR-CAS9 to incorporate genetic variations associated with psychiatric disorders, to enable more targeted drug testing [15]. Additionally, assembloids can be made by combining multiple organoids in order to study more complex cellular interactions. Cell lines and 3D cellular models are useful precisely because they are liminal—they are able to resemble human life closely enough without, for the most part, being seen as having potential moral status.^2^ At the same time, moral questions, such as the best model of consent required for their generation and the enduring personal connection donors may feel towards these entities, persist in relation to 3D models [16]. These concerns, as we will explore, have a long history and have been unresolved even in relation to immortalised cell lines.

On account of the complexity of these developments in cell culture, and the continuity of issues between 2D and 3D models, we believe it is useful to focus on immortalised cell lines, as a test case for establishing the utility of our liminal bioethics approach. Hence, we will examine the liminal status of cell lines over the next two sections, while also giving some consideration to the implications of this discussion for future study of more complex 3D models.

Despite being among the most well-known types of novel biological entities, immortalised cell lines are actually not well understood in relation to their ontological status or moral value. They have been used routinely in scientific research for decades, with the first immortalised cell line of human origin discovered in 1951—HeLa cells, which were cancer cells taken from the cervix of a young African-American woman named Henrietta Lacks. In the popular imagination, immortalised cell lines are sometimes confused with stem cell lines, which have generally received more media attention due to controversies over embryonic research. Although stem cells are also immortal, we will keep our investigation focused by referring only to somatic cells that have undergone transformation such that they proliferate indefinitely (i.e. are immortal) when we speak of ‘cell lines’. The exception to this rule is IPS cells, which are not part of the scope of this paper since they are somatic cells transformed into stem cells.

In this section we will first explore the sources of liminality in relation to categorising cell lines, before arguing why the gift-property distinction—though an important context for ethical discussion—is ultimately inadequate for articulating their liminality, despite being so prevalent in the literature.

### 3.1 Cell lines and human histories

Cell lines and human tissue culture more broadly have spawned a huge literature, with one key issue being whether informed consent is possible for cell lines or excised tissue that will be used in ways not previously foreseen or known by the original donor—or whether the donor should always be re-consented. This is particularly pertinent to cell lines given their immortality; recontacting donors may be impossible or impractical. The issue of consent has also been linked to legal discussions regarding the applicability of property rights to the human body. With the rise of genomics, there has also been renewed interest in cell lines from the perspective of privacy and genetic information.

It is fair to say that cell lines have not, for the most part, been treated as problematic in and of themselves, in the way that certain novel entities like embryoids or brain organoids might. This is no doubt a consequence of the traditional and somewhat narrow focus on the issue of moral status in bioethics, which has come at the expense of thinking about other kinds of value or other ways of conceptualising the status of novel biological entities.^3^ Inasmuch as cell lines have been an issue for bioethics, they have been an issue directly at the level of policy and practice, supported to some extent by theoretical discussions of property or privacy rights, but not by robust discussion about the ontological status of cell lines themselves.

Yet the interminable discussions regarding consent, such as those surrounding the revision of the US Common Rule [17], reflect an underlying difficulty shared with many other novel biological entities: Extraction, modification, and even commodification of human cells do not totally erase the real human histories behind them, and within them (e.g. in their DNA), and this is a key reason for their ambiguous status across our well-established categories. The exact importance of this continuing human element—is it merely incidental, or does it make the cell line a continuation of the donor’s personality, or is it more nuanced than either of these options—is an ontological issue that cannot be reduced to, but should inform, practical questions regarding consent.

We suggest that this issue requires careful liminal analysis. We should attend to the biological aspects of cell lines that connect, as well as distinguish, them from human biological life; and we should reflect on these biological aspects to help us make ethical sense of the human element of cell lines. Although the framework of property rights has been an important context for discussing various aspects of cell lines and their liminality, we contend that the narrow focus and conceptual presuppositions of property rights ultimately do not adequately capture what is at stake with cell lines.

### 3.2 Moving beyond property rights

Locating cell lines as “between gift and property” [18] would seem to be an obvious choice for articulating their liminality, given the dominance of the property framework in the ethics of human tissue biobanking. Indeed, cell lines are ambiguous in relation to the concept of property rights, but their ambiguity seems to point beyond this concept.

The question of property rights in the body has been pursued both in relation to and separate from human tissue biobanking. Where cell lines in particular are concerned, the starting point of discussions tends to be the Mo cell line, cultivated from cells taken from the spleen of John Moore, once again without the patient’s permission, as with HeLa cells. It was the subject of the *Moore v. Regents of the University of California* case, in which Moore petitioned unsuccessfully for his property rights in the cell line to be recognised—rights which would have allowed him a share in profits generated from the cell line [19]. It helps to see the *Moore* case in the context of two related cases, also in the US, involving human tissue samples that had been donated voluntarily: *Greenberg v. Miami Children’s Hospital Research Institute* [20] and *Washington University v. Catalona* [21]. Both cases related to whether donors had any continuing rights to control over the use of such samples, and in both cases the courts ruled against the donors. In *Catalona* the court ruled that unconditional transfer was the default for gifts of human tissue, while in *Greenberg* the court expressly declared that any property right in tissue samples “evaporates” when the samples are gifted voluntarily [22].

These cases attempt to pigeonhole cell lines and other human tissue into the gift side of the gift-framework dichotomy, but not without leaving behind some ambiguity. In *Moore*, the court took the view that Moore had no property rights in the cell line since he did not appear to possess the full range of rights associated with property in relation to those cells. This has been disputed for the simple and sensible reason that even with more conventional entities that possess property rights, we often see limits on some property-related rights, such as with prescription medicine where the right to disposal is usually circumscribed [23].

But it would be misleading to think that the court in the Moore case determined that there were no property rights *whatsoever* in the cell line; arguably, the real—if implicit—question was *who* had claim to those property rights. Indeed, the dissenting opinion in the case suggested that, were a rival medical institute to steal the cells, the court would recognise a violation of property rights in such an instance [19]. As Feldman points out, this leads to an incongruous situation where cell lines are in effect treated as property where research institutions are concerned, given that they enter into contractual agreements to transfer cell lines to other institutions, while the person in whose body those cells originated can have no claim to property rights [23].

It is easy to see, therefore, why cell lines have occupied an ambiguous position in relation to property rights. Consider what it would be like if patients had unambiguous property rights in relation to excised biological material. In theory, patients would be able to enter into commercial agreements for research performed on their cells or tissue, and they could set conditions that might give them continuing control over the research. But this is contingent on patients already knowing that their biological material is going to be valuable in some particular way that is of interest to a particular researcher, and this is not commonly the case. Scientists may need to study a range of samples before coming to conclusions about which samples are especially valuable for further study. To allow all kinds of conditions to be placed at the outset would hinder scientific research, and from this point-of-view one can understand why the court took the stance it did in *Moore*. On the other hand, treating cells and tissue as altruistic gift, at least as the default, seems too one-sided, in favour of the interests of scientific researchers. It is true that we do not *typically* have continuing interests in excised cells and tissue, but if researchers can suddenly acquire interests in samples that turn out to be valuable, it is hardly surprising that donors might also develop new interests in response to new information about material taken from their bodies. Donors might now be in a position to develop views on how their biological material should and should not be used, or at least feel morally invested in the research outcomes made possible by their donated samples.

Indeed, such interests and values that we see in our biological material point to a broader problem: The gift-property conceptual framework is ill-suited to capturing what is special about the body and its biological material to our identity and our values as humans. This problem is readily illustrated by looking at paradigmatic understandings of property and ownership. Wall, for instance, argues that property rights are best suited to items that are contingently owned—it is important to recognise something as owned because it may not be in other circumstances, but we do not ‘own’ our bodies contingently. This point is important because it has ethical and legal implications for how we remedy the loss of property. Where property is lost or damaged, we expect damages to substitute for the loss in some equivalent way. By contrast, when biological material is lost—say, an ovum or sperm stored for assisted reproduction—there is rarely any such compensation possible. The harm that is caused is of a different normative character, relating to intimate human relations or to our sense of bodily integrity, rather than to ownership [24]. So even if we wished to call our bodies ‘property’, we would be speaking about property in a derivative or metaphorical way, involving a different set of values from paradigmatic cases of property.

Focusing on the complexities of the human body itself, Herring and Chau have also argued that whereas the ideal entity for property rights is a “stable subject matter which can be controlled and fixed”, our bodies “are not static, consistent constructions, but constantly undergoing change and renewal”. We continually exchange bodily material—from innocuous bits of skin and hair to bodily fluids which may or may not be infected—when we meet people, rendering unrealistic any “claim to have rights of exclusive control over our bodies”, which would be typical of property rights [25].

It is telling that Björkman and Hansson, who defend a modified account of property rights in relation to the human body, say that the more important normative question is what rights a person has in relation to different types of biological material—whether they are called ‘property’ or ‘ownership’ is a secondary, terminological matter [26]. We do not dispute the importance of that normative question, but we suggest that conceptual categories do matter—an inadequate framing of an ambiguous entity can lead to important values being overlooked, as the *Moore, Catalona* and *Greenberg* cases show.

The question of property rights ultimately points beyond itself to considerations about biology and human identity. Landecker seems to suggest as much in her 1999 article, titled “Between Beneficence and Chattel”. Although in that article she examines intricacies of property law in relation to cell lines, the more important point she stresses is the way cell lines are both continuous and discontinuous with the originating human body. In the end, her analysis is concerned with less about what is the correct interpretation of property, as with how our understanding of the boundedness of the body has changed due to technological advances [18]. This seems instructive, as we turn to the biological characteristics of cell lines in order to more precisely articulate their conceptual liminality.

## 4 Cell Lines: Between Body Part And Organism

What features of the biology of cell lines might give us a clue as to a different framing for its liminality, especially in relation to human life? In this section, we suggest that the immortality of cell lines, understood more precisely in relation to biological categories, points us to the importance of the concept of ‘organism’. On this basis, we argue that the liminality of cell lines might be located between body part and organism, and explore the ethical implications of this framing.

### 4.1 Immortality and instability

Perhaps the most striking feature of cell lines is that they are immortal. But is it a genuine immortality? Immortality, in the proper sense of the word, is not simply about living longer than everybody else, but is about never dying—is that not an unusual feature in the natural world? Immortality is certainly unusual for *organisms*, and even the famously immortal species of jellyfish, *Turritopsis dorhnii*, still undergoes a life cycle, albeit repeatedly. By contrast, cell lines have no life cycle. In fact, far from having an orderly biological existence, they are often unstable genetically, with genetic drift leading to significant changes in the genomes of cell lines over time [27, 28].

In order to understand what the immortality of cell lines entails, we first need to be clear about the conceptual differences between speaking of organisms as ‘alive’ and speaking of individual cells or organs as ‘alive’. No doubt, body parts can exhibit a certain degree of independent ‘life’ for some time when taken out of the human body—their cellular functions can still continue under the right conditions, and so long as they have been kept ‘alive’ they could, with the right technology, be reintegrated into the same or another body. But their abilities of growth, tissue repair, fighting off infections, to name a few, would generally be limited. This stands in contrast to more stable, long-term self-renewal of an organism’s life.

Despite their impressive growth, cell lines are much more like an extracted body part than one might initially think. The idea of them as a *novel* entity has perhaps obscured their closer relation to human biology than the popular perception. Indeed, in the *Moore* case the inventive role of the scientists in turning Moore’s cells into a different entity altogether was a key argument in denying Moore’s property rights [18, pp. 204-206]. But a fact that has sometimes been forgotten in debates about cell lines is that, in the case of HeLa cells, Lacks’ cervical cells were not *transformed* by the scientists into an immortal state, even though that is sometimes how it is described. The discovery of HeLa cells was made possible by two things—unusual changes to cells in Lacks’ body caused by her cancer, which we know now to be linked to the Human Papillovirus (HPV) [29], and continuing efforts to perfect human tissue culture [30]. Scientists today deliberately use various viral oncogenes—which are responsible for tumour formation—to transform extracted somatic cells into immortalised cell lines [31], but this technique was not known in Lacks’ time. So the immortality of many cell lines, to be blunt, is related to the immortality of tumorigenicity, and tumours can be understood in simple terms as malformed human tissue.

### 4.2 Organisms and their parts

So even though we call cell lines immortal, they do not seem to be a kind of (artificially engineered) organism. But are they body parts? We suggest that cell lines are instead in between these two categories, and that this dichotomy helps to locate their liminality more precisely. But the initial considerations above, though grounded in biology, ultimately depend on what the concept of ‘organism’ means, and what its relationship to its body parts is. Hence, further conceptual analysis is required to help us better understand the liminality of cell lines.

‘Organism’, like ‘property’, is a concept that comes with its own paradigmatic meanings and presuppositions. Recent debates in the philosophy of biology make it clear that disputes over the concept of ‘organism’ are no mere terminological matter but are about explanatory power. In this context, two conceptual features of “organism” are of particular importance: Causal agency at the organismic level, and unity and intelligibility as a whole.

These two features are necessary for understanding how a robust conception of ‘organism’ can challenge genetic reductionism. The modern synthesis of evolutionary biology, which is still the dominant view of evolution, takes genes to be the main drivers of evolution, with evolutionary changes gleaned from changes in allele frequencies at the population-level. On this view, as Nicholson describes it, organisms are just an “interface” between the causal power of genes and the environment, but have “no autonomous agency of their own” [32]. But the term ‘organism’ is making a comeback, as more than just a folk term or as referring to mere aggregate of genes. Its importance for explaining phenomena is being re-asserted in the following ways.

Firstly, genetic reductionism is being challenged through attention to evidence, for example, of the way multiple phenotypes can result from the same genotype. Conversely, alterations at the molecular level do not necessarily result in corresponding alterations to organs, with organismic development appearing to have a “great degree of tolerance of abnormal gene expression” [33]. This paves the way for thinking about the organism as an explanatory tool for lower-level molecular activity. Genes, one might suggest, do not cause growth and development independently; they work as parts of a wider system called the organism.

Then, to make sense of that system, we need to think at a higher rather than lower level of complexity and organisation. As Noble and Noble point out, bodily functions such as circulation or renal flow can only be understood by thinking about the higher-level organisation of particular systems of the body, which regulate themselves through processes like heat exchange and through the use of membranes. DNA alone does not produce or guarantee the functioning of these systems; what matters is how the parts work together systematically, in interaction with each other and the external environment. The body as a whole functions similarly, maintaining an internal constant state (homeostasis) to some degree, but also dynamically interacting with the environment and optimising different functions at different times (homeorhesis) [34]. This leads us to consider the causal agency of the organism as a whole and not just the agency of individual parts. Perhaps the most striking example of organismic agency in evolutionary biology is niche construction—that is, cases where “the activities of organisms shape the selective environment they experience and thus modulate the selection pressures acting on them” [35]. This leads to a situation of reciprocal causation, where the effects of evolutionary pressures, manifested in dispositions to act in certain ways, also becomes the cause of such evolutionary outcomes. So the organism regulates and organises itself, adapts to the environment and adapts the environment to itself, which influences how its own species is selected and therefore how genotypes and phenotypes are passed down.

So to speak of an organism is to speak of autonomy—in a biological rather than narrowly moral sense—and to presuppose a higher level of organisation and unity which makes lower level processes intelligible. Body parts, as we have seen, do have a certain level of organisation and unity, but it is somewhat derivative. What is unique about the part-whole relationship of organisms, unlike that of artefacts, is that the whole is conceptually prior to its parts. As Nicholson puts it, “the parts of an organism only acquire their identities *qua* parts as the whole progressively develops from an originally undifferentiated yet already integrated system” [32, p. 354]. But it would be too simplistic to say that the direction of causality is just from whole to parts rather than parts to whole. Organisms give rise to their body parts, but body parts are needed to carry out the tasks required for the ongoing survival, adaptation, and growth of organisms. After all, development does not stop with all body parts being fully formed. Organisms are self-renewing, constantly losing and gaining new material—and this, as argued earlier, is also why the property paradigm does not fit the human body.

Where do cell lines come in? First of all, with a more robust view of organisms and their parts we can safely say that the immortality of cell lines is conceptually on a different level from the (im)mortality of organisms. The impressive growth of cell lines seems to give them a kind of quasi-organismic life or biological autonomy that body parts in culture do not have. On the other hand, their genetic instability seems to count against them being a new type of organism. More importantly, cell lines do not seem to exhibit any kind of higher level organisation or unity that gives intelligibility to their lower level process of continual growth. Hence their immortality is Sisyphean; they are stuck in a limbo of perpetual but purposeless growth.

But of course, cell lines do not simply exist in nature and happen to resemble body parts in some respects, and organisms in others. They are derived from body parts—from differentiated cells—and transformed and cultured. That is to say, they are derived from the ongoing process of self-renewal that organisms undergo, but are extracted from that process in an ambiguous way. When a body part, especially a whole organ, is extracted from a body and placed in culture, it still retains a recognisable identity as the part of a whole. The conceptual shift we propose to help understand the status of cell lines is that they are not an extracted part, but an extracted *process* or bundle of processes from the range of processes that keep an organism a self-renewing system. A process is more abstract than a part—a cell line is the process of growth extracted from its context. This, we contend, makes cell lines truly and persistently liminal, between body part and organism, since growth is an important process that ties the part-whole relationship together.

Before turning to consider the ethical implications of this analysis, let us briefly consider the implications for understanding 3D cell models such as organoids and assembloids. A key difference here is that, whereas on our analysis cell lines do not exhibit any meaningful organisation, organoids are marked by their self-organising properties, which allow them to be ‘reminiscent of an organ or organ region’ [4, p. 907]. Organoids themselves are also further categorised into ‘guided’ organoids, where exogenous factors are used to guide the self-organisation of the organoid into a specific organ region, and ‘unguided’ organoids which though self-organising will exhibit a higher diversity of cells [4, p. 908]. It is not hard to see why neural organoids have thus been described as being at the ‘interface of in vitro and in vivo neurobiology’ [36].

While it is beyond the scope of this paper to enter into a full analysis of the best conceptual framing needed for understanding the liminal status of organoids and associated entities such as assembloids, it seems likely that the conceptual shift of an extracted process rather than part will be helpful for understanding organoids. Whereas immortalised cell lines seem to predominantly extract the process of growth from the original organism, what organoids and assembloids extract from an organism is still more subtle: particular functions of an organ or organ region, ranging from neuro-immune interactions and cell migration [4, p. 909], to organ development and disease progression [37]. The self-organisation of organoids and assembloids undoubtedly makes them more self-contained and closer to body parts than cell lines are, and in this way they the framing of in vitro and in vivo thus seems apt for articulating their liminality. But crucially, organoids and assembloids are liminal because of their relatively limited functions, which exist in abstraction from the whole range of functions that properly integrate an actual organ into an organism. This liminality is persistent precisely because organoids are only understood, and indeed useful, relative to the organic whole from which they extract key functions.

### 4.3 Liminal ethics

Returning to cell lines, we must now examine what ethical significance our conceptual analysis of liminality holds in relation to the creation and use of cell lines. As we have already explained and demonstrated, the choice of conceptual categories is an intrinsically normative matter. Choosing ‘gift vs property’ as one’s dominant framework may lead to the neglect of certain values. But does ‘body part vs organism’ and the liminal space between shed light on ethical values or principles we might otherwise miss? What sort of correspondingly liminal ethical solutions might we offer?

First of all, it is worth mentioning that the framing of cell lines as between body part and organism is related to Hofmann’s suggested framing of novel biological entities as being “between thing and person”. Hofmann notes that human biological material, though not a person “is not merely a thing” either and “carries characteristic or information” of that person [9]. Useful as Hofmann’s framing may be in other, more general contexts, we suggest it is too broad to precisely articulate what is ontologically interesting about cell lines, especially given that they remain distant from the possibility of personhood. By contrast, the concept of ‘organism’ and its related underpinning has helped us better articulate the ontology of cell lines.

Although ‘organism’ is not an explicitly ethical term in the way that ‘person’ is in bioethics, the ‘human organism’ is surely a term with wider normative significance beyond the conceptual role in biology that we have already explored. Most importantly, the conceptual liminality we have identified provides a new, more precise way of articulating the continuity and discontinuity between cell lines and the originating human organism. The notion of a vital process rather than part being extracted and immortalised reminds us that cell lines retain some liminal traces of the organism from whose cells they were cultured, which should not be obscured by arguments about whether cell lines are new inventions or not. This could lend some support to the legitimacy of donor’s claims in exercising some continuing control over their cell lines. On the flipside, our conceptual analysis of organisms also suggests that we are not our DNA. As human organisms, we are bodily systems of which DNA is not a master blueprint. So beyond privacy concerns about genetic information, it is not DNA in itself that makes cell lines a continuation of the donor’s human identity. This might suggest that any rights to continuing control should have reasonable limits.

We suggest that the notion of being a process rather than a part being extracted offers some further ethical insight into how we might reason about what might be acceptable limits to any continuing control that donors might claim over cell lines. Consider the following analogy: Cell lines are akin to the letters, diary entries, novels, and paintings that we produce, which preserve and immortalise a particular thought process. Importantly, through technology such processes of human intelligence can be shared as fungible entities, beyond the original paper or canvas on which they are produced. In being shared and read or viewed by others, they often contribute to a greater sense of shared humanity, or at least help us understand other, often hidden facets of human life. It is, in fact, an analogous liminality that allows such artefacts—being neither a personal possession nor an individual person—to play this role in human life.

Cell lines, we argue, ought to be seen in a similar way. Their ability to contribute to greater scientific understanding of humanity depends on the ability of scientists to readily share and access their information (e.g. in genomics), or indeed the cell lines themselves (e.g. for disease-modelling and vaccine testing). In line with their quasi-organismic autonomy, a greater deal of freedom for their movement and use must be allowed, compared with body parts which are more strongly associated with their owners or donors, for which we place greater restrictions surrounding their sale and commodification. But there should be limits to this free use. Hence, the ethics of cell lines seems to be somewhere between full autonomy based on consent and strict deontological prohibition.

Analogously again, when we disseminate our ideas through art and literature, we recognise that despite their continuity with our own life, our artistic products must be allowed to go around society somewhat autonomously. It does not mean there are no limits. For example, the French Intellectual Property Code grants artists a moral right to the protection of an artwork’s integrity [38, L121-1],^4^ such that even the new owner of an artwork must not mutilate or misuse her new possession. Pixabay, a large provider of copyright-free images, states in its terms and conditions that images featuring a recognisable person must not be used in an “immoral or illegal way”—ranging from obscene or libellious use to even usage in a political context [39], which presumably is intended to prevent recognisable persons from being associated with political movements unknowingly. These are ways in which we accommodate and address the somewhat liminal human element of artworks, which do not arise from considerations of property rights, but on the moral rights or interests associated with that human element.

Likewise, in dealing with cell lines—and arguably this applies to other human tissue samples as well—there should not be a presumption of unconditional use, even in cases where broad consent has been given. Instead, there should be a presumption that practices which are or might be generally disapproved of by society or which could be constituted as disrespectful to human life—for example, the use of cells for cloning or the creation of human-animal hybrids—should not be carried out using cell lines and human tissue, or at least not without further re-consent. This also suggests that tiered consent, where the donor can opt out of specific types of further use listed on the consent form, is ethically preferable to broad consent, at least if it can be implemented practically.

How does one determine what practices might constitute disrespect to human life in the context of using cell lines? The difficulty, of course, is that this is less readily determined than what might count as a disrespectful use of copyright-free human images (pornography, violence, etc.). Perhaps the final, broad insight that liminality suggests is that the ethical principles or solutions we offer in relation to liminal entities should always be seen as being constantly in transition or negotiation. This means we must resist the usual attempt to find once-and-for-all, definitive policy solutions and accept that this is not possible with some entities. We would certainly expect the ethics of what counts as disrespectful use of images and artworks to always be in a state of flux, being at the intersection of different interests and values, compared with what is disrespectful to human beings or their possessions, which though changeable over time is more stable. In a similar fashion, the ethics of cell lines and human tissue samples must be continually subject to consultation [40], empirical study of social attitudes, and rigorous analysis of what might plausibly cause disrespect even to a liminal element of human life.

## 5 Conclusion

In this article, we have offered a new method for analysing the status of novel biological entities and its ethical implications, and demonstrated how it might be applied in practice. We believe that the overall method we propose can be easily accepted and its value for bioethical reflection is not difficult to understand, given that liminality is a real conceptual phenomenon and attempts to resolve liminality by assimilation to established concepts does not always produce satisfactory results. The true difficulty lies in the detailed conceptual analysis that is required for assessing the suitability of different conceptual categories, and here we stress that there is no automatic method of discovery. Even when settling on one pair of concepts that seems to articulate the liminality of an entity particularly well, we must remain open to the possibility that other liminal framings may be possible and may give insight into other neglected ethical aspects.

It is also clear from our comparatively brief discussion of organoids in the previous section that there is potential for further bioethical study of liminality to be pursued. We see our study of relatively simpler immortalised cell lines as a kind of ‘proof of concept’, which can be extended to more complex cases. Given the sheer diversity of organoids, both in relation to organs they model and to types of organoids, it may be that there are multiple types of liminal statuses involved in organoid technology. These may in turn raise different ethical concerns—for example, with neural organoids typically being more contentious than other types of organoids, owing to the moral importance of the brain and the potential for consciousness. The framing of being at the interface of in vitro and in vivo, though initially compelling, may thus require further precision.

## Footnotes

1 Svenaeus applies the term “sobjects” primarily to extracted body parts such as blood, bones or organs, rather than to novel entities.

2 One notable exception concerns the possibility of neural organoids acquiring the capacity for consciousness.

3 In response to this problem, van Till et al. propose going beyond moral status in evaluating novel entities such as organoids, and using a more fine-grained taxonomy to evaluate moral value, which comprises instrumental, intrinsic, symbolic, and relational values [41].

4 We thank Jonathan Herring for suggesting this example.
